# Menopause: an opportunity to optimize health and well being for people with HIV

**DOI:** 10.1097/COH.0000000000000944

**Published:** 2025-04-21

**Authors:** Shema Tariq

**Affiliations:** a Institute for Global Health, University College London; b Central and North West London NHS Foundation Trust, London, UK

**Keywords:** ageing, comorbidities, HIV, menopause, women's health

## Abstract

**Purpose of review:**

Menopause, defined as 12 months without menstruation, is a complex biopsychosocial transition. This review synthesizes current knowledge on menopause in individuals living with HIV, highlighting its clinical significance, research gaps, and approaches for optimizing care.

**Recent findings:**

Women and people with ovaries with HIV may experience menopause earlier, and with more severe vasomotor, mood, and musculoskeletal symptoms compared to people without HIV. Increasing severity of symptoms is associated with reduced quality of life and poorer engagement in HIV care. Additionally, estrogen depletion combined with HIV increases the risk of cardiometabolic disease and osteoporosis. Biomarkers like AMH have shown promise for assessing ovarian reserve in this population, but current evidence remains inconclusive. Menopause remains under-recognized in HIV care, with low rates of menopausal hormone therapy use and limited provider confidence in menopause management.

**Summary:**

Addressing menopause in people with HIV is vital for improving quality of life, supporting engagement in HIV care, and reducing comorbidity risk. Integrated and holistic care models, peer support, and focused research are essential to meet the needs of this growing population and close existing gaps in care.

## INTRODUCTION

**FIGURE 1:**
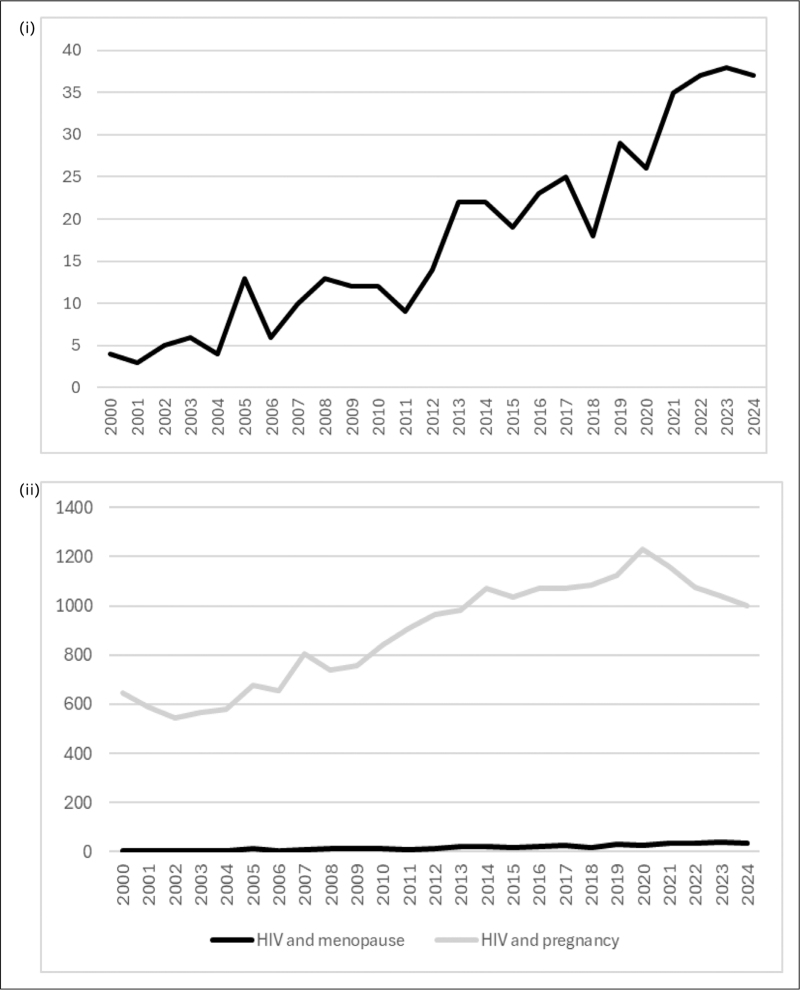
Number of publications/year on (i) HIV and menopause and (ii) HIV and menopause vs. HIV and pregnancy (based on PubMed search 10/03/25). (i) Number of publications on HIV and menopause, 2000–2025. (ii) Number of publications on HIV and menopause vs. HIV and pregnancy, 2000–2025.

Improved access to effective antiretroviral therapy (ART) and an increase in HIV diagnoses among older individuals have contributed to a growing number of people aged 50 and over living with HIV. Estimates suggest that the global population in this age group rose from 5.4 million in 2015 to 8.1 million in 2020 [1]. Consequently, menopause (defined as the absence of periods for 12 months) has become a key area of focus in HIV care and research. Over the past 25 years, the number of published articles on HIV and menopause has steadily risen, however HIV and pregnancy remains the dominant focus of attention in HIV and women's health (Fig. 1). 

**Box 1:**
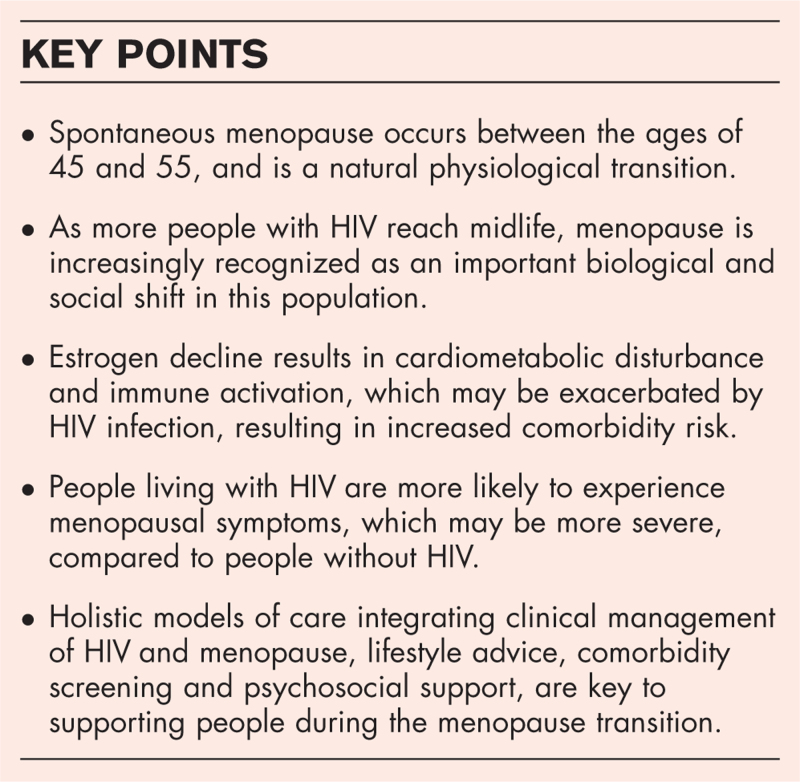
no caption available

This paper aims to synthesize current data on menopause in individuals living with HIV, offering a broad overview, identifying research gaps, and proposing strategies to optimize clinical care. I aim to be gender inclusive, recognizing that menopause is also experienced by transgender men and gender nonbinary people. I will therefore refer to individuals, and/or women and people with ovaries. However, when discussing studies conducted only in cisgender women, I will refer to women.

## BROAD OVERVIEW OF MENOPAUSE

Menopause is a biopsychosocial shift, marked by the cessation of ovarian function (often indicated by the cessation of menstruation) and the irreversible loss of reproductive potential [2]. For the majority of women and people with ovaries, menopause is spontaneous, occurring between the ages of 45 and 55 as a result of ovarian ageing [3]. People also experience iatrogenic menopause as a result of bilateral oophorectomy and/or medical treatment.

Perimenopause, the phase preceding the last menstrual period, is characterized by fluctuating estrogen and progesterone levels, leading to a wide range of symptoms and menstrual irregularity. Estrogen and progesterone levels eventually stabilize and remain low as people reach menopause and postmenopause, at which point many symptoms subside [4]. The median duration of vasomotor symptoms (including hot flushes and night sweats), which are experienced by 80% of people during menopause, is 7.4 years [5].

The most prevalent symptoms during the menopause transition are vasomotor symptoms, mood changes, sleep disruption and urogenital symptoms (including vaginal dryness) [6]. Many of these are a direct consequence of the loss of estrogenic effects on systems that require estrogen for optimal function, for instance the brain and urogenital tract. Estrogen also plays a pivotal role in cardiometabolic and musculoskeletal health, leaving individuals at increased risk of comorbidities such as osteoporosis and cardiovascular disease (CVD) after menopause [2].

## AGE AT MENOPAUSE

Age at menopause is a marker of biological ageing and risk of age-related conditions in people with ovaries. Understanding variation in age at menopause also supports individuals and healthcare providers to correctly identify menopausal symptoms and to seek appropriate management. Non-iatrogenic menopause occurs between the ages of 45 and 55, with a meta-analysis of studies across 24 countries reporting a median age at menopause of 48.8 years [7]. However, there is geographical variation in age at menopause, ranging from 47.2 years in Latin America to 51.3 years in Australia [7].

Some studies have reported an earlier age at menopause among women living with HIV [8–10,11^▪▪^]. A systematic review on the association of HIV status with age at menopause found that five out of seven studies reported an earlier age at menopause among women living with HIV (between 46 and 50 years). However, they were limited by the lack of biochemical confirmation of menopause, meaning other causes of prolonged amenorrhoea could not be excluded [12]. Furthermore, the association between HIV status and age at menopause is likely to be confounded by sociodemographic variables such as ethnicity, substance use, hepatitis co-infection, and body mass index (BMI) [13].

The association between HIV status and both early menopause (menopause aged 40–45 years) and premature ovarian insufficiency (POI, menopause aged <40 years) is clearer. Van Ommen *et al.'s* systematic review found that all six studies reporting prevalence of early menopause or POI, showed an increased prevalence in women with HIV [12]. Factors associated with early menopause and POI include lower educational status, hepatitis C co-infection, younger age at HIV diagnosis, and smoking [10,14,15].

## BIOMARKERS OF OVARIAN ACTIVITY IN HIV

Studies on age at menopause in people living with HIV are complicated by the increased prevalence of prolonged amenorrhoea unrelated to menopause [16–18]. Menstrual irregularity is associated with substance use, concomitant medication (such as psychotropic medication), chemotherapy, low BMI, smoking and hepatitis B co-infection [16–18]. Biomarkers of ovarian activity may therefore provide more diagnostic certainty.

### Anti-Müllerian Hormone

Anti-Müllerian hormone (AMH) is a biomarker of ovarian reserve, reflecting the remaining quantity of an individual's egg supply [19]. Unlike other reproductive hormones, AMH remains relatively stable throughout the menstrual cycle, making it a more reliable indicator of ovarian function. A recent study involving 462 women, including 256 living with HIV, found that AMH levels were lower in women living with HIV aged <35 compared to their HIV-negative counterparts, suggesting a potential negative impact of HIV on ovarian reserve [20]. However, a systematic review examining ovarian reserve biomarkers states that current evidence on the relationship between AMH and HIV is inconclusive, with sociodemographic factors playing a significant confounding role [21]. An analysis of the Women's Interagency HIV Study cohort demonstrated that AMH was highly predictive of age at final menstrual period in women living with HIV [22]. However, it is important to exercise caution as the diagnostic value of AMH in individual patients has yet to be established [23].

### Follicle-stimulating hormone

During menopause, follicle-stimulating hormone (FSH) levels rise significantly as the ovaries produce less estrogen and progesterone, leading to reduced negative feedback on the pituitary gland [3]. This increase in FSH is a key marker of menopause; however, during perimenopause FSH can fluctuate considerably, limiting its reliability. As a result, diagnosis of peri- or postmenopause should be a clinical diagnosis in individuals aged over 45 years in the general population, with FSH not recommended unless someone is aged 45 years or under [24]. This guidance can also be applied to people living with HIV aged over 45 who are virologically suppressed on ART [25].

## MENOPAUSAL SYMPTOMS

Menopausal symptoms are common in individuals with HIV, as they are in people without HIV [26]. The prevalence of menopausal symptoms in women living with HIV has been reported to be over 70%, with between 30% and 55% reporting severe symptoms [27,28]. The most prevalent symptoms include joint and muscle pain, mood changes, hot flushes, fatigue and sleep disruption [27–30]. Factors associated with increased severity of menopausal symptoms in women living with HIV include perimenopause (compared with pre and postmenopause), substance and/or alcohol use, depression, and financial and/or food insecurity [27–29,31].

There is some evidence to suggest that people living with HIV experience more severe symptoms [29,32,33]. Vasomotor symptoms, reduced sexual function, low mood, and anxiety are more commonly reported by women with HIV during the menopause transition than their HIV-negative counterparts [34–37]. However, as with age at menopause, we must be aware of the confounding effect of factors such as ethnicity, socioeconomic status, and substance use [38,39].

Increasing severity of menopausal symptoms is not only associated with reduced health-related quality of life (QoL) among living with HIV [29,40], but is also linked to poor engagement in HIV care as measured by adherence to ART [41–44] and missed clinic visits [42].

## THE IMPACT OF MENOPAUSE ON HIV PROGRESSION AND RESPONSE TO ART

Estradiol has important immunomodulatory effects [45], although its effects on HIV latency and progression remain under-investigated. Of note, a study of virally suppressed postmenopausal women has found an expansion in the inducible HIV RNA+ reservoir [46]. This reservoir is rich in replication-competent virus, which has important implications for future cure strategies. There is increasing interest in the impact of menopause on the gut microbiome in individuals living with HIV; two studies have demonstrated changes in the gut microbiome that are likely to be driven by estrogen depletion [47,48^▪▪^]. These changes may be linked to increased cardiometabolic risk and immune activation [48^▪▪^,^49^,^50^].

Data on the impact of menopause on response to ART are especially sparse. However, there is no evidence to suggest that menopause has a detrimental effect on response to ART, ART tolerability, or ART safety [51–53].

## MENOPAUSE AND COMORBIDITY RISK IN HIV

Estrogen depletion during menopause significantly affects cardiometabolism and bone density, increasing the risk of medical conditions beyond ageing alone [3]. This risk is further amplified in the context of HIV and ART, which independently contribute to some conditions. Women living with HIV have been found to have an increased prevalence of multimorbidity at all ages, compared to women without HIV and men living with HIV [54]. The prevalence of multimorbidity increases significantly from the age of 50, with perimenopause associated with an increased burden of non-AIDS-related comorbidities [54,55]. Despite this, menopausal status and comorbidity risks remain under-documented among women living with HIV during and after the menopause transition [56,57^▪^,^58^].

### Cardiovascular impact

Estrogen plays a crucial cardioprotective role by regulating lipid metabolism, improving endothelial function, reducing oxidative stress, and promoting vasodilation [3]. Its decline during menopause leads to increased cardiovascular disease (CVD) risk, which is further exacerbated by HIV. Data suggest that HIV has a greater impact on CVD risk in women than men [58], with women exhibiting accelerated subclinical atherosclerosis [59^▪▪^]. Additionally, current CVD risk scores likely underestimate risk in women living with HIV, as they do not account for menopausal status [60^▪▪^]. This may lead to suboptimal risk-based decisions, reinforcing gender disparities in CVD outcomes.

### Metabolic impact

Estrogen depletion can result in adverse changes in lipid metabolism, increased risk of insulin resistance and diabetes, and weight gain and altered fat deposition. Among women living with HIV, obesity and overweight are highly prevalent, and is more common in those of Black ethnicities [61]. The menopause transition has also been linked to increased waist circumference in women living with HIV [62]. Additionally female sex and older age are particularly associated with weight gain on ART. Recent data have shown that women who switch to an integrase strand transfer inhibitor during peri or postmenopause experience faster weight gain and greater increases in waist circumference compared to premenopausal women [63^▪▪^,^64^]. Furthermore, women with metabolic dysfunction-associated steatohepatitis are at higher risk of faster progression to fibrosis during perimenopause [65^▪^].

### Bone density impact

Finally, estrogen plays an essential role in bone health, inhibiting bone resorption, stimulating bone formation and regulating calcium absorption [3]. HIV infection and menopausal status are each independently associated with decreased bone mineral density (BMD) [66,67]. Among women living with HIV, the rate of BMD decline accelerates during the menopause transition [68,69], with some evidence that midlife women living with HIV have a higher fracture rate than those without HIV [70]. Factors associated with low BMD and fractures in midlife women living with HIV include older age, low BMI, longer duration of menopause smoking and prior fracture [70,71].

## MANAGEMENT OF MENOPAUSE IN PEOPLE WITH HIV

In this paper I have outlined the high prevalence of menopausal symptoms in people living with HIV, the impact symptoms have on QoL and engagement in HIV care, and the increased risk of comorbidities as a result of the synergistic effects of estrogen depletion and HIV. Studies have shown that healthcare providers in both primary care and HIV clinics lack confidence in identifying and managing menopause in people living with HIV [57^▪^,^72^]. In a Canadian cohort, fewer than half of women living with HIV aged 35 years or older had ever discussed menopause with their healthcare provider, even though almost all were regularly engaged in HIV care [73].

Menopause presents an opportunity to optimize the health and well being of people living with HIV. Menopausal hormone therapy (MHT, also known as hormone replacement therapy or HRT) is recommended for the treatment of vasomotor and mood-related symptoms [24]. The preferred regimen is transdermal estrogen, with either micronized progesterone or Mirena IUS as endometrial protection for those with a uterus, due to a lower risk of venous thromboembolism and potentially breast cancer than nonbody identical preparations.

Despite a small but increased risk of breast cancer, MHT has been shown to reduce symptoms and improve QoL. Although we currently lack long-term data on efficacy and safety of MHT in people with HIV, there is no reason to think the benefits and risks would differ from the general population. Most modern antiretrovirals don’t interact with MHT, and should there be interactions, they can be managed by adjusting doses of MHT according to symptoms and side effects. Yet MHT use remains very low, with only 8.7–11.8% of people with HIV using it, and even fewer (5.6%) using vaginal estrogen [8,73,74].

In recent years, menopause guidelines have been developed specifically for people living with HIV [75^▪▪^], and integrated HIV and menopause services have been introduced [76]. These guidelines emphasize proactively assessing menopausal status and symptoms (using a validated tool such as the Menopause Rating Scale [77]); reviewing overall health and comorbidity risks; encouraging lifestyle changes; optimizing ART; discussing MHT options; and providing ongoing support (Table 1). This support should ideally include peer support tailored to the needs of women and people with ovaries ageing with HIV, such as the UK-based GROWS Programme (Women with HIV GRowing Older Wiser and Stronger) [78].

**Table 1:** A holistic approach to managing menopause in people living with HIV

1. Annual assessment of menstrual pattern in all women and people with ovaries, and annual assessment of menopausal symptoms using a validated tool such as the Menopause Rating Scale in all women and people with ovaries aged ≥40
2. Assessment of comorbidity risk (such as low bone mineral density and cardiovascular risk) as per national HIV monitoring guidelines
3. Enquire about routine cervical and breast screening history
4. Ascertain contraceptive need
5. Routine enquiry about domestic violence
6. Provide advice on lifestyle optimization including weight bearing exercise, healthy diet (with referral to a dietician if required), smoking cessation, minimizing alcohol and other substance use, and sleep hygiene
7. Optimise antiretroviral therapy to minimize drug interactions with menopausal hormone therapy (MHT) and reduce toxicity e.g. switching off TDF and/or efavirenz
8. If aged ≥40 consider initiating a statin if no contraindications
9. If MHT indicated (for vasomotor and/or mood symptoms), discuss risks and benefits
10. First line MHT should be transdermal estradiol, with micronized progesterone (or Mirena IUS) if the individual has a womb, titrated according to symptoms and potential drug interactions^a^ (see https://www.hiv-druginteractions.org/)
11. If MHT declined or contraindicated, consider cognitive behavioural therapy for vasomotor symptoms, or nonhormonal treatments such as venlafaxine
12. If genitourinary symptoms are present advise topical vaginal estradiol and vaginal moisturisers
13. Provide tailored written information on HIV and menopause^b^, and psychological and/or peer support^c^
14. Liaise with primary care for ongoing management

a www.hiv-druginteractions.org/.

b https://sophiaforum.net/women-with-hiv-growing-older-wiser-and-stronger-grows/.

c https://sophiaforum.net/guide-to-menopause-for-women-living-with-hiv/.

## CONCLUSION

Increasing numbers of people living with HIV are reaching menopausal age. The intersection of HIV and menopause presents unique challenges that require careful consideration. Individuals living with HIV experience more severe menopausal symptoms, as well as an increased risk of comorbidities. Despite this, menopause remains under-recognized and under-managed in this population.

Although research on menopause in the context of HIV is growing, it still lags behind other areas such as HIV and pregnancy. Future research priorities include the validation of menopause symptom scales in populations living with HIV, long term safety and efficacy data on MHT in people living with HIV, the potential role of MHT as primary prevention for osteoporosis and/or cardiometabolic disease, data on new non-hormonal treatments such as fezolinetant and ospemifene, and more studies from high prevalence areas, where the majority of the global population of women ageing with HIV reside.

Addressing menopause in people with HIV offers a valuable opportunity to improve quality of life, support healthy aging, reduce long-term health risks and support engagement in care. Greater awareness, education for healthcare providers, integrated care models, and tailored peer support are essential to ensure that people living with HIV are supported through the menopause transition, and that existing gender health inequities are not further amplified.

## Acknowledgements

*The author received no assistance in writing this paper.*

*The author received funding for the PRIME (Positive Transitions Through the Menopause) Study from the National Institute for Health Research (NIHR) (PDF-2014-07-971), a UCL/Wellcome Institutional Strategic Support Fund Flexible Support Award (204841/Z/16/Z) and a British HIV Association (BHIVA) Research Award (2017).*

*The author has received consultancy fees and speaker honoraria from Gilead Sciences, Janssen-Cilag, and ViiV Healthcare.*

### Financial support and sponsorship

*None.*

### Conflicts of interest

*There are no conflicts of interest.*

## REFERENCES AND RECOMMENDED READING

Papers of particular interest, published within the annual period of review, have been highlighted as:

▪ of special interest
▪▪ of outstanding interest
